# Metastatic malignant melanoma from anorectum presenting as an isolated breast tumor: A case report and literature review

**DOI:** 10.1097/MD.0000000000031174

**Published:** 2022-10-14

**Authors:** Xiaokang Yi, Hao Chen, Ankang Wang, Feng Liu, Hua-Mao Zhang

**Affiliations:** a Department of Hepatobiliary Surgery, The Dujiangyan Medical Center, Chengdu, Sichuan, China; b Department of Liver Surgery, West China Hospital, Sichuan University, Chengdu, Sichuan, China; c Department of General Surgery, Nanchong Central Hospital, The Second Clinical College of North Sichuan Medical College, Nanchong, Sichuan, China; d Department of Thyroid Breast Vascular Surgery, The Dujiangyan Medical Center, Chengdu, Sichuan, China.

**Keywords:** breast, case report, melanoma, metastasis, surgery

## Abstract

Anorectal malignant melanoma regularly exhibits a biological aggressive behavior which is metastasizing to lung, bone, brain or other organs and tissues early in the course of the disease. Compared with melanoma in the other parts of the body, anorectal malignant melanoma is relatively rare. Metastatic to the breast tissue from anorectal malignant melanoma or from other extra-mammary tumors are very rare.

**Patient concerns:**

We report the case of a 65-year-old female who suffering from anorectal malignant melanoma and implemented complete surgical resection. Two years later, a space-occupying lesion in the outer upper quadrant of the right breast was observed on a chest CT.

**Diagnosis:**

The right breast was excised, and breast metastasis of anorectal malignant melanoma was histologically confirmed.

**Interventions:**

Radical mastectomy of the right breast was performed, and no lymph nodes or other metastases were observed.

**Outcomes:**

The patient’s operative course was uneventful. The patient completely recovered and transfers to the oncology department for further treatment.

**Lesson:**

The patient presented with an isolated breast tumor. Duo to Malignant melanoma could mimic many kind of poorly differentiated tumors, it is difficult to diagnose accurately, especially when it appears as an isolated mammary tumor. Because of the treatment measures and prognosis between malignant melanoma and breast cancer are entirely different.

## 1. Introduction

Malignant melanoma (MM) which routinely occurs on the human’s skin and mucous membranes is a highly malignant tumor brought about by the excessive proliferation of atypical melanocytes. The global incidence of melanoma is rising.^[1]^ Compared with melanoma in the other parts of the body, anorectal malignant melanoma is relatively rare. The incidence of mucosal malignant melanoma in the yellow race Mongolian is higher than white Caucasian. In addition, breast metastasis of anorectal malignant melanoma or other extra-mammary tumors is not common and represent ~1.3 to 2.7% of cases.^[2,3]^ The present study report a case of breast metastasis of anorectal malignant melanoma and review previously published case reports on this rare disease.

## 2. Case presentation

**Figure 1.**
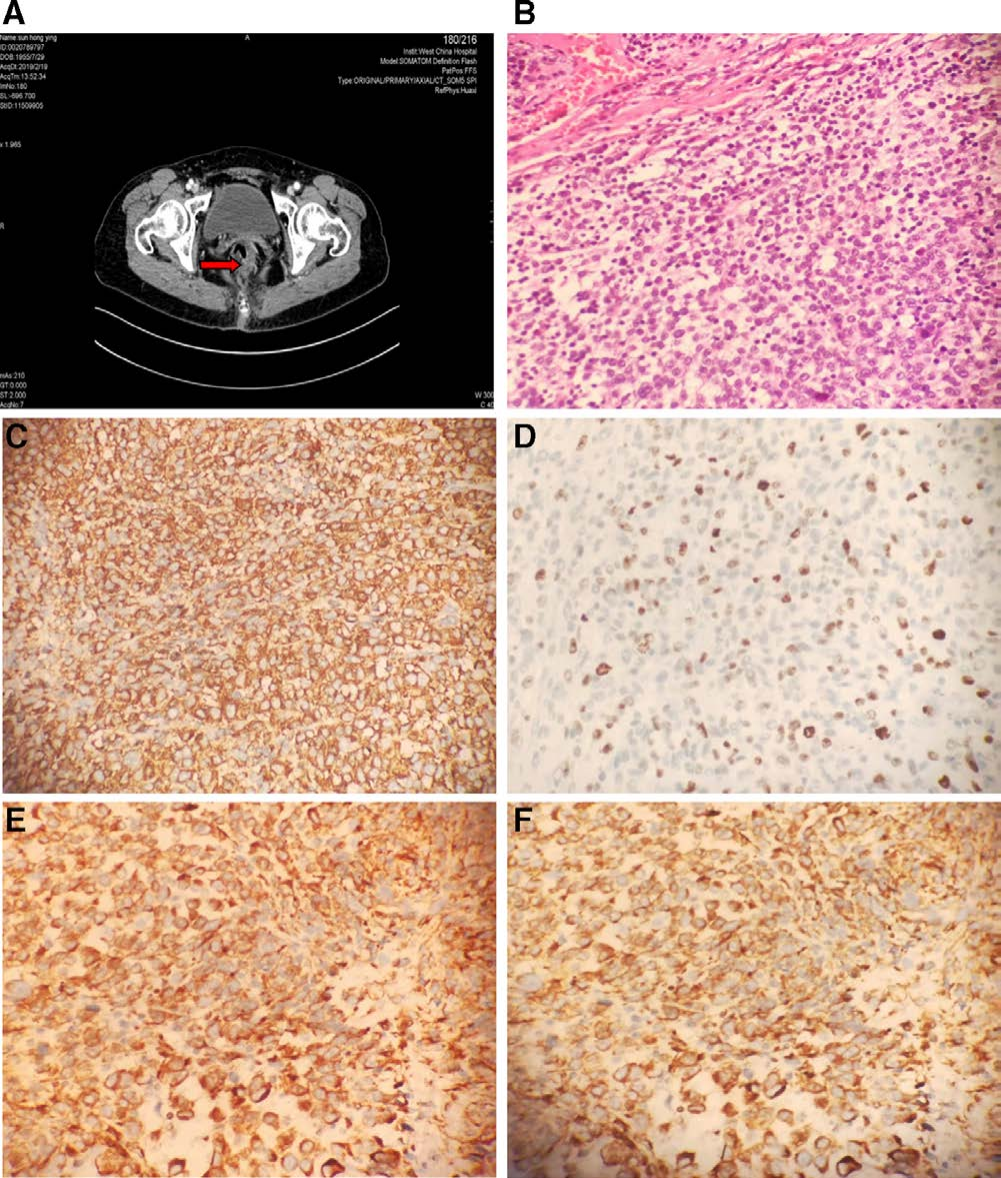
Examination results of anorectal malignant melanoma. Notes: (A) The abdominal CT revealed a space-occupying lesion in the rectum. (B) Smaller tumor cells with a diffuse distribution are observed in the anorectal tumor; hematoxylin–eosin stain. (C) Tumor cells showing positive staining for HMB-45; immunohistochemistry staining. (D)Tumor cells showing positive staining for Melan-A; immunohistochemistry staining. (E) Tumor cells showing positive staining for S-100; immunohistochemistry staining. (F) Tumor cells showing positive staining for vimentin; immunohistochemistry staining.

A 63-year-old woman with a negative family history of cancer was diagnosed with “Anorectal malignant tumor” at the West China Hospital of Sichuan University (Sichuan, China) in February 2019 due to “intermittent blood in the stool for 2 months”. After admission, the laboratory assessment illustrated a hemoglobin (Hb) level of 108 g/L (normal range: 115–150 g/L). The abdominal CT scan discovered that the intestinal wall of the lower rectum-anal canal area is thickened and eccentric, mainly on the left and the posterior parts, local lumen is constricted, and some of the surrounding lymph nodes are enlarged (Fig. 1A). The possibility of malignant tumor was considered. She underwent surgical resection and histology revealed anorectal malignant melanoma (Fig. 1B). Immunohistochemical staining was performed and says HMB-45 (+, Fig. 1C), Melan-A (+, Fig. 1D), S100 (+, Fig. 1E), Vimentin (+, Fig. 1F), PCK (−), P16 (−).

**Figure 2.**
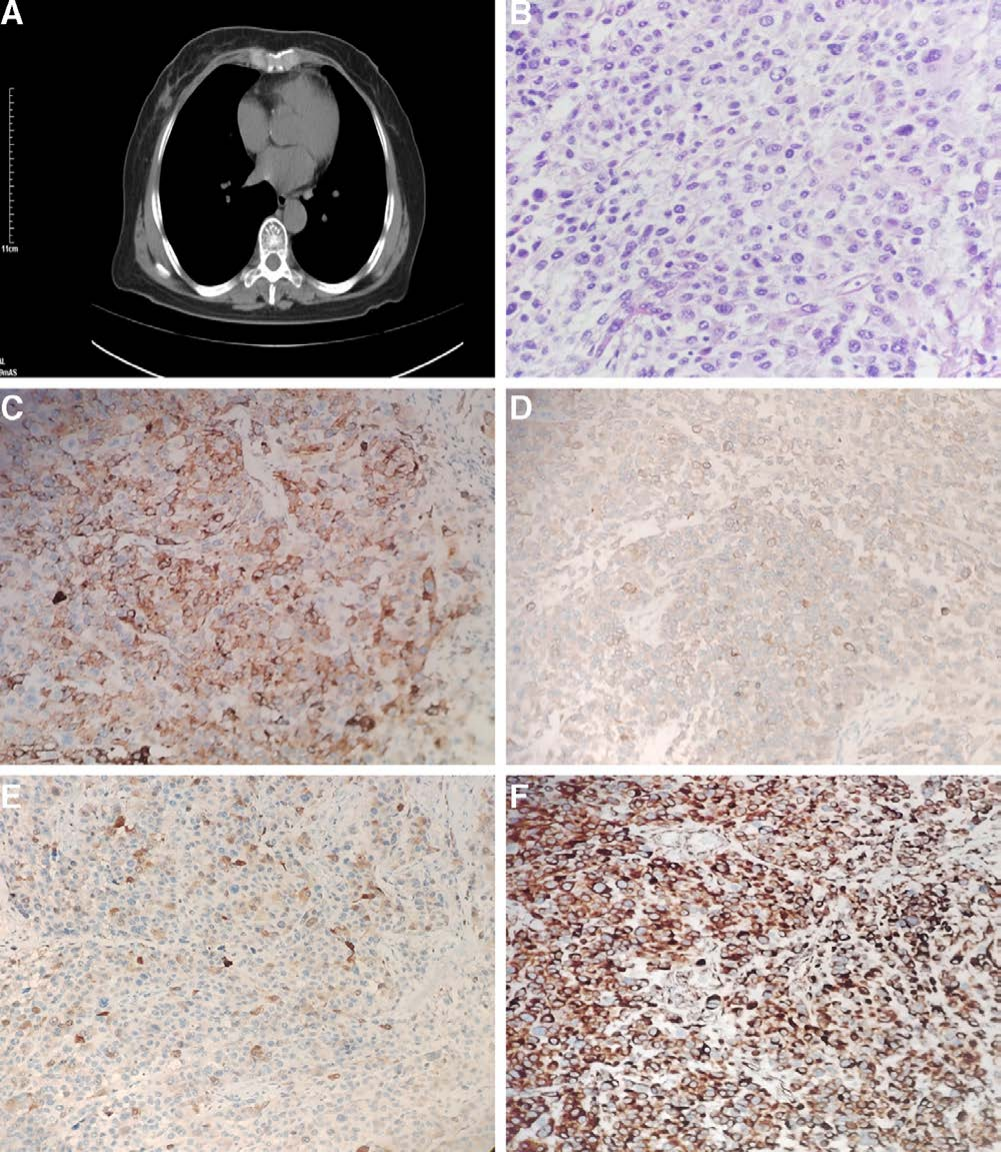
Examination results of anorectal metastatic malignant melanoma. Notes: (A) CT scan of the chest revealed A space-occupying lesions sized 2.3 cm is seen in the outer upper quadrant of the right breast. (B) Metastatic tumor in the outer upper quadrant of the right breast showing morphological findings consistent with those of the anorectal melanoma; hematoxylin–eosin stain. (C) Metastatic tumor cells showing positive staining for HMB-45; immunohistochemistry staining. (D) Metastatic tumor cells showing positive staining for Melan-A; immunohistochemistry staining. (E) Metastatic tumor cells showing positive staining for S-100; immunohistochemistry staining. (F) Metastatic tumor cells showing positive staining for vimentin; immunohistochemistry staining.

She remained systematically healthy until 29 months later when, during a CT scan done for staging purposes at the Dujiangyan Medical Center (Sichuan, China), unfortunately, a newly visible lump with unclear boundaries was noted in the outer upper quadrant of the right breast, which showed 2.3 cm nodules, considered as neoplastic lesions (Fig. 2A). In order to further diagnose, the patient underwent a core needle biopsy of the suspicious masses in the right breast under ultrasound guidance. The results revealed that biopsies were consistent with malignant tumor. No other metastases were noted by the PET/CT. Then we performed a modified radical mastectomy and histologically confirmed that it was the metastatic malignant melanoma (Fig. 2B). Immunohistochemical staining shows HMB-45 (+, Fig. 2C), Melan-A (+, Fig. 2D), S100 (+, Fig. 2E), Vimentin (+, Fig. 2F), CK (−), CK7 (−), CD34 (−), WT-1 (−), which confirmed that it is the anorectal malignant melanoma metastatic to the breast.

## 3. Discussion

Most breast malignancies are derived from a primary cancer of the breast tissue. Metastasis of the breast is an uncommon presentation of any breast mass which has been reported that metastasis presenting as a breast mass constitutes 0.5% to 2% of all breast malignancies.^[4]^ Anorectal melanoma regularly occurs close to the dentate line. Due to the extreme aggressiveness of the tumor and the abundantly lymphatic vessels near the dentate line, local spread and distant metastases may occur in the early stage of the disease, with an extremely rare metastasis rate to the breast, resulting in a 5-year overall survival of <20%.^[2,5–7]^ In this study (Table 1), a systematic review of the PubMed database was performed using the following keywords: (Anorectal [Title]) and (Melanoma [Title]) and (Breast [Title]), it yielded only 3 articles, describing 3 cases of anorectal melanoma metastasis to the breast reported from 1999 to 2016,^[8–10]^ the patient in the present study represents the fourth case reported in the English literature.

**Table 1:** Data summary of the case series.

Case number	Sex	Age (yrs)	History	Primary surgery	Time tometastasis (mo)	Site	Management of breast lump	Adjuvant therapy after metastasis	Time interval between breast metastasis and death (mo)	References
1	F	66	Anorect al	RR	29	Right upper outer	RR	NP	NA	This case
2	F	55	Anorectal	RR	13	Left upper outer	NP	Biotherapy	5	9
3	F	59	Anorectal	RR	4	Left upper outer	RR	NP	42	10
4	F	55	Anorectal	NP	3	Left	NP	NP	2.5	11

F = female, NA = not available, NP = not performed, RR = radical resection.

Anorectal malignant melanoma seems to be a disease detected in older individuals. Tumors mostly involve the upper outer quadrant, which is in line with the prevalence of breast cancer. According to the current statistics, the ailing population is all women. Therefore, we suspect that this disease is related to estrogen. In 1984, Lee’ research showed that melanoma cells express estrogen receptors^[11]^; it was previously reported that breast metastasis of anorectal malignant melanoma mostly occurred at the premenopausal stage. An article reported 15 patients with melanoma metastatic to the breast, of which 93% occurred before menopause.^[12]^ Another retrospective study involving 27 cases also reported that 70% of patients were in the premenopausal stage.^[2]^ Arora and Robinson proposed a direct role for estrogen in promoting metastatic spread,^[12]^ but it seems to be contrary to the statistical results in this research.

The pathological diagnosis of MM is usually based on the following factors, such as epithelioid or spindle cell morphology; cell discohesion; eosinophilic cytoplasm with fine, dustlike or coarse, granular pigment; and vesicular nuclei with inclusion-like macronucleoli.^[4]^ Therefore, in the appropriate clinical context, the diagnosis of MM can be given with a relative degree of confidence, based on pathological examination. Nonetheless, the evidence, a number of clinical and pathological features, may lead to inappropriate treatment in cases of anorectal malignant melanoma appears as an isolated breast tumor, especially when the patients or the attending doctors neglect to convey a previous history of melanoma or when medical history has no contribution, and the error eventually may led the patients to the abyss of death. Therefore, it is extremely important to distinguish the anorectal malignant melanoma to the breast in a large number of malignant diseases.

Metastasis to the breast usually indicated a poor prognosis. Feng et al^[8]^ reported that eight of 15 patients who died within 1 year after metastasis to the breast. As Ravdel et al^[2]^ reported, the median survival time of 27 patients with melanoma metastases to the breast was 12.9 months. Most melanoma patients who had metastasized to the breast already have local spread and distant metastases, which usually include subcutaneous nodules, lung, brain, liver and so on.^[2]^ However, the patient in this study, an isolated breast tumor was found, and no other sites of metastases were found by PETCT. Radical surgical resection is the first choice of treatment, and radiotherapy, chemotherapy, immunotherapy and other treatments are the auxiliary schemes. This is a great opportunity for radical surgery.

As we all know, immunohistochemical staining is the golden indicator of diagnosis among all the evidence. It disclosed negativity for cytokeratins, CD45, desmin, estrogen receptor, and progesterone receptor, whereas antibodies against other antigens such as S100 protein, Vimetin, HMB-45, and Melan-A, were strongly reactive.^[13]^ Actually, duo to MM to the breast could simulate a variety of poorly differentiated carcinoma and many rare types of tumors, its differential diagnosis is a complicated task. The first need to be considered is the poorly differentiated breast carcinoma. Yet it is just one of the imitators need to be ruled out. Furthermore, data from Bacchi Carlos point that the leiomyosarcoma, liposarcoma, lymphoma, and medullary carcinoma also can be simulated by MM.^[13]^ The breast of primary leiomyosarcoma is rare, but not unexpectedly, reported.^[14,15]^ It is worth noting that primary lymphoma is uncommon in the breast, but it is not accidental. It has a wide range of clinical and microscopic manifestations, including anaplastic large cell lymphoma, a well-known simulant of epithelial tumors.^[16–18]^ Interestingly, it has also been noted that positivity for S100 protein in cases of primary leiomyosarcoma of the breast.^[19,20]^

It is a very rare condition that metastasis of tumors to the breast, for patients with a history of melanoma or other malignant tumors, the possibility of metastasis should be considered when a mass is found in the breast tissue. The present study found that the anorectal malignant melanoma metastatic to the breast, of which 100% occurred in postmenopausal women, which is inconsistent with mainstream views, and it required further research to explain this phenomenon.

## Author contributions

Xiao-Kang Yi Feng Liu and Hua-Mao collected the information, followed the patient, and wrote the paper;Hao Chen and Hua-Mao Zhang revised the paper; Feng Liu was the patient’s doctor in charge; All authors read and approved the final manuscript.

**Conceptualization:** Xiaokang Yi.

**Data curation:** Xiaokang Yi, Ankang Wang, Hua-Mao Zhang.

**Formal analysis:** Xiaokang Yi, Ankang Wang.

**Investigation:** Xiaokang Yi, Feng Liu.

**Methodology:** Xiaokang Yi.

**Project administration:** Hao Chen, Feng Liu.

**Resources:** Xiaokang Yi, Hao Chen, Hua-Mao Zhang.

**Writing – original draft:** Xiaokang Yi.

**Writing – review & editing:** Xiaokang Yi.

**Writing – review & editing:** Hua-Mao Zhang.
